# The COVID-19 Pandemic Has Had Negative Effects on Baseline Clinical Presentation and Outcomes of Patients with Newly Diagnosed Treatment-Naïve Exudative AMD

**DOI:** 10.3390/jcm10061265

**Published:** 2021-03-18

**Authors:** Enrico Borrelli, Marco Battista, Giovanna Vella, Domenico Grosso, Riccardo Sacconi, Lea Querques, Ilaria Zucchiatti, Francesco Prascina, Francesco Bandello, Giuseppe Querques

**Affiliations:** Department of Ophthalmology, University Vita-Salute, IRCCS Ospedale San Raffaele, 20132 Milan, Italy; borrelli.enrico@hsr.it (E.B.); battista.marco@hsr.it (M.B.); giovanna.vella28@gmail.com (G.V.); grosso.domenico@hsr.it (D.G.); sacconi.riccardo@hsr.it (R.S.); querques.lea@hsr.it (L.Q.); zucchiatti.ilaria@hsr.it (I.Z.); prascina.francesco@hsr.it (F.P.); bandello.francesco@hsr.it (F.B.)

**Keywords:** COVID-19, retina, neovascular AMD, outcome

## Abstract

Purpose: To investigate whether the coronavirus disease 2019 (COVID-19) pandemic-associated postponement in care had effects on the baseline clinical presentation of patients with newly diagnosed treatment-naïve exudative neovascular age-related macular degeneration (AMD). Methods: We included the first 50 consecutive patients referred within the COVID-19 pandemic with a diagnosis of treatment-naïve exudative neovascular AMD. Two groups of fifty consecutive patients with newly diagnosed neovascular exudative AMD presenting in 2018 and 2019 (control periods) were also included for comparisons. Results: Baseline visual acuity was statistically worse in patients referred during the COVID-19 pandemic period (0.87 ± 0.51 logarithm of the minimum angle of resolution (LogMAR)) as compared with both the “2019” (0.67 ± 0.48 LogMAR, *p* = 0.001) and “2018” (0.69 ± 0.54 LogMAR, *p* = 0.012) control periods. Data on the visual function after a loading dose of anti-vascular endothelial growth factor (VEGF) was available in a subset of patients (43 subjects in 2020, 45 in 2019 and 46 in 2018, respectively). Mean ± SD best corrected visual acuity (BCVA) at the 1-month follow-up visit after the third anti-VEGF injection was still worse in patients referred during the COVID-19 pandemic (0.82 ± 0.66 LogMAR) as compared with both the “2019” (0.60 ± 0.45 LogMAR, *p* = 0.021) and “2018” (0.55 ± 0.53 LogMAR, *p* = 0.001) control periods. On structural optical coherence tomography (OCT), the maximum subretinal hyperreflective material (SHRM) height and width were significantly greater in the COVID-19 pandemic patients. Conclusions: We demonstrated that patients with newly diagnosed treatment-naïve exudative neovascular AMD referred during the COVID-19 pandemic had worse clinical characteristics at presentation and short-term visual outcomes.

## 1. Introduction

The development of macular neovascularization (MNV) is a frequent cause of vision decrease in age-related macular degeneration (AMD) patients [1,2]. The anti-vascular endothelial growth factor (VEGF) treatment is currently considered as the gold standard in eyes with exudative neovascular AMD.

While the anti-VEGF treatment proved to be effective for preventing disease progression and improving vision in patients with neovascular AMD [3,4,5], a number of patients still do not respond ideally to this treatment. As an example, both the ANCHOR (Anti-VEGF Antibody for the Treatment of Predominantly Classic Choroidal Neovascularization in AMD) [6] and MARINA (Minimally Classic/Occult Trial of the Anti-VEGF Antibody Ranibizumab in the Treatment of Neovascular AMD) [7] trials revealed that ~10% of patients have a significant loss of visual acuity in the first 24 months of treatment after baseline. A number of studies have identified baseline clinical characteristics associated with worse treatment outcomes [8,9,10,11,12,13]. Among these, an early detection and prompt treatment were demonstrated to improve the visual outcome [14].

Outpatient care has been significantly revised during the coronavirus disease 2019 (COVID-19) pandemic. As an example, in order to decrease the possibility of virus transmission, providers have postponed elective and preventive visits, and outpatient assessments have been limited to more urgent care [15,16,17,18,19]. At the same time, visits have been delayed by patients to avoid being exposed. Our group has recently quantified the decrease in in-person visits and intravitreal procedure volumes during the COVID-19 pandemic, and we displayed that outpatient care in AMD patients was the most impacted during this period [20].

Assuming that patients experiencing a longer delay between their first symptoms of exudative AMD and their first anti-VEGF injection have a lower likelihood of ameliorating vision after treatment, Ref. [21] the COVID-19-related contraction in outpatient volume and the consequent delay to treatment might have resulted in significant repercussions on the clinical presentation of patients with treatment-naïve neovascular AMD.

Therefore, the purpose of our study was to quantify the effects of postponed care on clinical characteristics and outcomes of subjects with newly diagnosed treatment-naïve exudative neovascular AMD. In details, we assessed the baseline functional and anatomic features of these patients. Additionally, we analyzed the short-term visual outcomes after a 3-month loading dose of anti-VEGF. Our results may provide a valuable insight into elements influencing outcomes in real-life emergency settings. Furthermore, our data may be beneficial in handling AMD patients during successive waves of the COVID-19 outbreak or in other emergency settings.

## 2. Methods

The San Raffaele Ethics Committee was notified about this observational study, as for Italian legislation. This study adhered to the tenets of the Declaration of Helsinki and Health Insurance Portability and Accountability Act. Participants gave informed consent to be included in the study.

### 2.1. Study Participants

Subjects 50 years of age and older with newly diagnosed treatment-naïve neovascular AMD were enrolled from a medical retinal practice (Medical Retina and Imaging Unit) at the San Raffaele Scientific institute in Milan, Italy. In detail, the first 50 consecutive patients referred after 9 March 2020 (the date in which the first Italian COVID-19 lockdown was established) were included. Enrolled patients had a complete ophthalmologic examination including structural optical coherence tomography (OCT) that was performed with a Heidelberg Spectralis HRA + OCT (Heidelberg Engineering, Heidelberg, Germany) device. OCT macular imaging included 19 B-scans, each of which comprised 24 averaged scans, covering approximately 5.5 × 4.5 mm area centered on the fovea. OCT scans were included in presence of a minimum signal strength of 25, as advised by the manufacturer [22].

Exclusion criteria for analyzed eyes included: (i) any history of previous anti-VEGF injections; and (ii) any evidence of maculopathy secondary to causes other than AMD.

In order to understand whether treatment-naïve exudative neovascular AMD patients enrolled during the COVID-19 pandemic had different clinical characteristics at presentation, we also included two groups of fifty consecutive patients with newly diagnosed neovascular exudative AMD presenting in 2018 and 2019 (control periods) for comparison.

### 2.2. OCT Grading

Structural OCT images were graded for qualitative features previously proposed as suggestive of exudative disease activity, Refs. [23,24,25] including presence of subretinal fluid (SRF), intraretinal fluid (IRF), and subretinal hyperreflective material (SHRM). Structural OCT images were reviewed by two independent and experienced readers (EB and MB). Based on previous studies, Refs. [26,27] a lesion was graded as present if the reader had a more than 90% confidence that it was recognizable in at least 1 B-scan. Graders later met to compare level of agreement, and disagreements were resolved by open adjudication to yield a single assessment for each case. In those cases in which the graders did not agree on a single consensus result, the final decision was made by the senior author (GQ). A single reviewer (GV) also reviewed the OCT images for quantitative features, including: (i) maximum SRF height and width; and (ii) maximum SHRM height and width. These measurements were performed by calipers on structural OCT as previously described [28,29].

### 2.3. Statistical Analysis

Statistical calculations were performed using Statistical Package for Social Sciences (version 20.0, SPSS Inc., Chicago, IL, USA). Continuous variables were compared by conducting independent samples *t*-tests. Statistical significance of the differences for qualitative variables was assessed using Fisher’s exact test.

A *p* value < 0.05 was determined to be statistically significant.

## 3. Results

Clinical characteristics of analyzed subjects are summarized in Table 1. Of the 150 patients (150 eyes) with treatment-naïve newly diagnosed neovascular AMD included, 50 were consecutively enrolled in 2020, whereas 50 patients were included in 2019 and 50 patients had baseline visits in 2018. Time to complete the necessary enrollment of 50 consecutive patients was 207 days in 2020, 93 days in 2019, and 104 days in 2018.

On structural OCT, no differences in terms of qualitative characteristics (presence of IRF, SRF, and SHRM) were detected among the study periods (Table 1). Considering only patients with evidence of SHRM, the maximum SHRM height and width were significantly greater in the COVID-19 pandemic patients (245.2 ± 228.2 μm and 2316.2 ± 461.9 μm, respectively) as compared with both the “2019” (126.6 ± 122.6 μm and 760.3 ± 128.5 μm, *p* = 0.016 and *p* = 0.009, respectively) and “2018” (148.9 ± 166.2 μm and 947.1 ± 151.7 μm, *p* = 0.045 and *p* = 0.016, respectively) periods.

Similarly, considering only patients with OCT evidence of SRF, the maximum SRF height and width were slightly greater in “2020” patients (1828.3 ± 1045.4 μm and 180.5 ± 108.6 μm, respectively) in comparison with patients referred in the two previous years (1250.9 ± 772.4 μm and 163.6 ± 52.3 μm in 2019, 1356.1 ± 711.3 μm and 166.1 ± 87.9 μm in 2018, respectively), although this difference did not reach the statistical significance (*p* = 0.080 and *p* = 0.513 for 2019 vs. 2020, *p* = 0.120 and *p* = 0.607 for 2018 vs. 2020, respectively).

Baseline best corrected visual acuity (BCVA) was statistically worse in patients referred during the COVID-19 pandemic period (0.87 ± 0.51 logarithm of the minimum angle of resolution (LogMAR)) as compared with both the “2019” (0.67 ± 0.48 LogMAR, *p* = 0.001) and “2018” (0.69 ± 0.54 LogMAR, *p* = 0.012) control periods (Table 1). Of the 50 patients included in this analysis during the COVID-19 pandemic, 31 had dry AMD in the fellow eye (unilateral neovascular AMD group), while 19 were affected by previously treated neovascular AMD in the fellow eye (bilateral neovascular AMD group). At baseline, BCVA was slightly better in the bilateral neovascular AMD group (0.83 ± 0.49 LogMAR) than in the unilateral neovascular AMD group (0.90 ± 0.53 LogMAR), although this difference did not reach statistical significance (*p* = 0.215).

Data on the visual function after a 3-month loading dose of anti-VEGF was available in a subset of patients (43 subjects in 2020, 45 in 2019, and 46 in 2018, respectively). Mean ± SD BCVA at the 1-month follow-up visit after the third anti-VEGF injection was still worse in patients referred during the COVID-19 pandemic (0.82 ± 0.66 LogMAR) as compared with both the “2019” (0.60 ± 0.45 LogMAR, *p* = 0.021) and “2018” (0.55 ± 0.53 LogMAR, *p* = 0.001) control periods.

## 4. Discussion

In this study we assessed whether the postponement in diagnosis and therapy secondary to the COVID-19 pandemic had an impact on patients with newly diagnosed treatment-naïve exudative neovascular AMD. Overall, we proved that patients referred during the COVID-19 pandemic had different baseline clinical characteristics as compared with subjects presenting in the two previous control years. In addition, short-term visual outcomes were significantly worse in these patients, as compared with patients referred in the two pre-pandemic control periods. Our findings suggest that the COVID-19-related deferral in in-person visits was significantly associated with worse baseline clinical characteristics in these patients.

In a recent study, Ref. [20] our group proved that the COVID-19 outbreak caused a significant decrease in in-person visits and intravitreal procedures at a referral medical retina practice. Importantly, this reduction was more pronounced in older patients, especially in those subjects affected by AMD [20]. Many factors were speculated to be implicated in the reduction in outpatient volume. First, ophthalmology departments applied novel risk-stratification protocols to reduce personnel and patient risks [15,16,19,30]. These guidelines were also aimed at reducing the person-to-person transmission by deferring non-urgent visits. Second, patients were also limited in crossing borders between regions, and this may have further restricted their capability to attend visits. Third, many patients also procrastinated visits because of a fear of COVID-19 exposure. In the present study, the time to complete the necessary enrollment of 50 consecutive patients was superior in the pandemic period, this suggests a significant COVID-19-related delay for patients’ presentation.

In a successive study, [31] we investigated the impact of COVID-19-related delayed care on the visual and anatomic outcomes of subjects with neovascular AMD under a pro re nata (PRN) approach. In detail, we enrolled 100 consecutive patients that had a follow-up visit (pandemic visit-V_0_) between 9 March 2020 and 12 June 2020 (during and immediately after the first COVID-19 outbreak wave in Italy). In this study cohort, functional and anatomical findings from this visit were compared with those obtained from the two preceding visits (pre-pandemic visits: V_-1_ and V_-2_). In the latter study, we demonstrated that clinical characteristics of enrolled patients were significantly worse at the pandemic visit, as visual acuities were diminished and OCT signs of exudation were more prevalent at this assessment. Based on these findings, we concluded that the COVID-19 pandemic-related postponement in patient care was significantly associated with worse short-term outcomes in AMD patients under PRN therapy.

We add to the literature by reporting the impact of delayed care secondary to the COVID-19 pandemic on the clinical presentation of newly diagnosed treatment-naïve exudative neovascular AMD patients. In our study cohort of patients referred for exudative neovascular AMD during the COVID-19 pandemic, the visual acuity was significantly worse as compared with patients referred in the two control periods. Holz and colleagues [32] reviewed 2227 patients with neovascular AMD and receiving anti-VEGF treatment in clinical practice. The latter study of real-life anti-VEGF therapy demonstrated that a lower baseline visual acuity was significantly associated with worse outcomes in these patients. Delay to treatment was demonstrated to be responsible for irreversible visual acuity deterioration in treatment-naïve neovascular AMD patients [14,33,34]. Therefore, our results may suggest that the COVID-19-related delay in care may have significant and irreversible effects on the visual outcomes of these patients. These interpretations are further confirmed by our post hoc analysis on patients with longitudinal data. Indeed, visual acuity was still worse in the pandemic group after the loading anti-VEGF therapy, as compared with the two control periods.

Although differences were not statistically significant, our analysis seems also to suggest that patients with neovascular AMD in the fellow eye had a better visual acuity at presentation. This is probably related to the fact that patients who had already experienced neovascular AMD in the fellow eye are less prone to delay the initial visit and start therapy, as previously suggested [35,36].

Several studies have suggested that structural OCT imaging may be a valuable and reliable tool for determining baseline predictive factors of treatment response in eyes with exudative neovascular AMD. Subretinal hyperreflective material or SHRM is visualized using structural OCT as hyperreflective material located below the neuroretina [12,37]. This OCT feature may represent various tissue forms, including fluid, fibrin, blood, scar, and vessels [12,25,37,38]. Of note, SHRM was shown to be an important morphologic biomarker for neovascular AMD, as both its presence and characteristics were associated with poorer visual outcomes in treatment-naïve neovascular AMD eyes [12,39,40]. In our study cohort, in those patients with SHRM at the baseline visit, the lesion size was significantly greater in patients referred during the COVID-19 pandemic. Using structural OCT, Kumar and colleagues [41] analyzed 170 patients with a diagnosis of treatment-naïve exudative neovascular AMD and a follow-up of 24 weeks. The authors of this study measured the baseline (pre-treatment) SHRM characteristics on structural OCT images and correlated these values with visual outcomes at the 12-week and 24-week follow-up visits. They demonstrated that baseline SHRM size was significantly associated with worse visual acuities at follow-up visits. Furthermore, SHRM decreases in size with anti-VEGF therapy [12] and thus a delay in treatment may facilitate a growth in SHRM size. Taken together, these data may suggest that the COVID-19-related delay in care among patients with newly diagnosed exudative neovascular AMD might have caused a growth in SHRM size, and this may significantly impact on the final outcome of these patients.

Limitations of this report include the analysis of a single community setting in northern Italy, which to date is one of the most affected regions worldwide. Furthermore, we were not able to provide a multimodal imaging description of our patients as our study cohort was not homogeneously imaged with the same modalities. As a consequence, we did not report on associations between the nature of SHRM and outcomes. The strengths of our study include its design and the large sample size of consecutively enrolled patients.

In conclusion, in a tertiary referral retina unit, the COVID-19-related postponement in care proved to be significantly associated with worse baseline clinical characteristics in patients with newly diagnosed treatment-naïve exudative neovascular AMD. Although the COVID-19 pandemic is limited in time, these findings may help broaden our knowledge regarding the management of these patients in an emergency setting. Finally, our results may help eye doctors to estimate the chance of vision deterioration associated with visits’ postponement and treatment delay in patients with treatment-naïve exudative AMD.

## Figures and Tables

**Table 1:** Characteristics of analyzed patients in the three study periods.

	2020	2019	2018
**Number of patients (eyes), *n***	50 (50)	50 (50)	50 (50)
**Gender, *n* (%)**	23 (46.0%) males	22 (44.0%) males	25 (50.0%) males
	27 (54.0%) females	28 (56.0%) females	25 (50.0%) females
		1.0 ^a^	1.0 ^b^
**Mean age (years),** **mean ± SD**	80.7 ± 6.6	81.1 ± 5.9	79.9 ± 7.0
		1.0 ^a^	1.0 ^b^
**BCVA (LogMAR), mean (SD)**	0.87 ± 0.51	0.67 ± 0.48	0.69 ± 0.54
		0.001 ^a^	0.012 ^b^
**Type of MNV, *n* (%)**	30 type 1 (60.0), 9 type 2 (18.0), 11 type 3 (22.0)	27 type 1 (54.0), 10 type 2 (20.0), 13 type 3 (26.0)	35 type 1 (70.0), 6 type 2 (12.0), 9 type 3 (18.0)
		1.0 ^a^	1.0 ^b^
**OCT evidence of intraretinal fluid, *n* (%)**	32 (64.0)	30 (60.0)	31 (62.0)
		1.0 ^a^	1.0 ^b^
**OCT evidence of subretinal fluid, *n* (%)**	30 (60.0)	33 (66.0)	32 (64.0)
		1.0 ^a^	1.0 ^b^
**OCT evidence of SHRM, *n* (%)**	35 (70.0)	32 (64.0)	32 (64.0)
		1.0 ^a^	1.0 ^b^

*n*: number of eyes; BCVA: best corrected visual acuity (logMAR (logarithm of the minimum angle of resolution)); MNV: macular neovascularization; SD: standard deviation; OCT: optical coherence tomography; SHRM: subretinal hyperreflective material. ^a^ comparison versus V_-1_; ^b^ comparison versus V_-2_.

## Data Availability

The data used to support the findings of this study are available from the corresponding author upon request.

## Additional Information

Francesco Bandello is a consultant for: Alcon (Fort Worth, Texas, USA), Alimera Sciences (Alpharetta, Georgia, USA), Allergan Inc (Irvine, California, USA), Farmila-Thea (Clermont-Ferrand, France), Bayer Shering-Pharma (Berlin, Germany), Bausch And Lomb (Rochester, New York, USA), Genentech (San Francisco, California, USA), Hoffmann-La-Roche (Basel, Switzerland), Novagali Pharma (Évry, France), Novartis (Basel, Switzerland), Sanofi-Aventis (Paris, France), Thrombogenics (Heverlee, Belgium), Zeiss (Dublin, USA). Giuseppe Querques is a consultant for: Alimera Sciences (Alpharetta, Georgia, USA), Allergan Inc (Irvine, California, USA), Amgen (Thousand Oaks, USA), Bayer Shering-Pharma (Berlin, Germany), Heidelberg (Germany), KBH (Chengdu; China), LEH Pharma (London, UK), Lumithera (Poulsbo; USA), Novartis (Basel, Switzerland), Sandoz (Berlin, Germany), Sifi (Catania, Italy), Sooft-Fidea (Abano, Italy), Zeiss (Dublin, USA). The other authors have no disclosures.

## References

[1] Spaide R.F., Jaffe G.J., Sarraf D., Freund K.B., Sadda S.R., Staurenghi G., Waheed N.K., Chakravarthy U., Rosenfeld P.J., Holz F.G. (2020). Consensus Nomenclature for Reporting Neovascular Age-Related Macular Degeneration Data. Ophthalmology.

[2] Borrelli E., Sarraf D., Freund K.B., Sadda S.R. (2018). OCT angiography and evaluation of the choroid and choroidal vascular disorders. Prog. Retin. Eye Res..

[3] Martin D.F. (2018). Evolution of Intravitreal Therapy for Retinal Diseases—From CMV to CNV: The LXXIV Edward Jackson Memorial Lecture. Am. J. Ophthalmol..

[4] Keane P.A., Sadda S.R. (2011). Development of Anti-VEGF Therapies for Intraocular Use: A Guide for Clinicians. J. Ophthalmol..

[5] Solomon S.D., Lindsley K., Vedula S.S., Krzystolik M.G., Hawkins B.S. (2014). Anti-vascular endothelial growth factor for neovascular age-related macular degeneration. Cochrane Database Syst. Rev..

[6] Brown D.M., Michels M., Kaiser P.K., Heier J.S., Sy J.P., Ianchulev T. (2009). Ranibizumab versus Verteporfin Photodynamic Therapy for Neovascular Age-Related Macular Degeneration: Two-Year Results of the ANCHOR Study. Ophthalmology.

[7] Rosenfeld P.J., Brown D.M., Heier J.S., Boyer D.S., Kaiser P.K., Chung C.Y., Kim R.Y. (2006). Ranibizumab for Neovascular Age-Related Macular Degeneration. N. Engl. J. Med..

[8] Ying G.-S., Huang J., Maguire M.G., Jaffe G.J., Grunwald J.E., Toth C., Daniel E., Klein M., Pieramici D., Wells J. (2013). Baseline Predictors for One-Year Visual Outcomes with Ranibizumab or Bevacizumab for Neovascular Age-related Macular Degeneration. Ophthalmology.

[9] Regillo C.D., Busbee B.G., Ho A.C., Ding B., Haskova Z. (2015). Baseline Predictors of 12-Month Treatment Response to Ranibizumab in Patients With Wet Age-Related Macular Degeneration. Am. J. Ophthalmol..

[10] Chae B., Jung J.J., Mrejen S., Gallego-Pinazo R., Yannuzzi N.A., Patel S.N., Chen C.Y., Marsiglia M., Boddu S., Freund K.B. (2015). Baseline Predictors for Good Versus Poor Visual Outcomes in the Treatment of Neovascular Age-Related Macular Degeneration With Intravitreal Anti-VEGF Therapy. Investig. Ophthalmol. Vis. Sci..

[11] Ying G.-S., Maguire M.G., Pan W., Grunwald J.E., Daniel E., Jaffe G.J., Toth C.A., Hagstrom S.A., Martin D.F. (2018). Baseline Predictors for Five-Year Visual Acuity Outcomes in the Comparison of AMD Treatment Trials. Ophthalmol. Retin..

[12] Willoughby A.S., Ying G.-S., Toth C.A., Maguire M.G., Burns R.E., Grunwald J.E., Daniel E., Jaffe G.J., Williams D.F., Beardsley S. (2015). Subretinal Hyperreflective Material in the Comparison of Age-Related Macular Degeneration Treatments Trials. Ophthalmology.

[13] Segal O., Barayev E., Nemet A.Y., Geffen N., Vainer I., Mimouni M. (2016). Prognostic value of hyperreflective foci in neovascular age-related macular degeneration treated with bevacizumab. Retina.

[14] Lim J.H., Wickremasinghe S.S., Xie J., Chauhan D.S., Baird P.N., Robman L.D., Hageman G., Guymer R.H. (2012). Delay to Treatment and Visual Outcomes in Patients Treated with Anti-Vascular Endothelial Growth Factor for Age-Related Macular Degeneration. Am. J. Ophthalmol..

[15] Parravano M., Borrelli E., Costanzo E., Sacconi R., Varano M., Querques G. (2020). Protect Healthcare Workers and Patients from COVID-19: The Experience of Two Tertiary Ophthalmology Care Referral Centers in Italy. Ophthalmol. Ther..

[16] Borrelli E., Sacconi R., Querques L., Zucchiatti I., Prascina F., Bandello F., Querques G. (2020). Taking the right measures to control COVID-19 in ophthalmology: The experience of a tertiary eye care referral center in Italy. Eye.

[17] Corradetti G., Corvi F., Nguyen T.V., Sadda S.R. (2020). Management of Neovascular Age-Related Macular Degeneration during the COVID-19 Pandemic. Ophthalmol. Retin..

[18] Shmueli O., Chowers I., Levy J. (2020). Current safety preferences for intravitreal injection during COVID-19 pandemic. Eye.

[19] Iovino C., Caporossi T., Peiretti E. (2020). Vitreoretinal surgery tip and tricks in the era of COVID-19. Graefes Arch. Clin. Exp. Ophthalmol..

[20] Borrelli E., Grosso D., Vella G., Sacconi R., Querques L., Zucchiatti I., Prascina F., Bandello F., Querques G. (2020). Impact of COVID-19 on outpatient visits and intravitreal treatments in a referral retina unit: Let’s be ready for a plausible “rebound effect”. Graefes Arch. Clin. Exp. Ophthalmol..

[21] Holz F.G., Tadayoni R., Beatty S., Berger A., Cereda M.G., Hykin P., Staurenghi G., Wittrup-Jensen K., Altemark A., Nilsson J. (2016). Key drivers of visual acuity gains in neovascular age-related macular degeneration in real life: Findings from the AURA study. Br. J. Ophthalmol..

[22] Huang Y., Gangaputra S., Lee K.E., Narkar A.R., Klein R., Klein B.E.K., Meuer S.M., Danis R.P. (2012). Signal Quality Assessment of Retinal Optical Coherence Tomography Images. Investig. Ophthalmol. Vis. Sci..

[23] Lee S.Y., Stetson P.F., Ruiz-Garcia H., Heussen F.M., Sadda S.R. (2012). Automated Characterization of Pigment Epithelial Detachment by Optical Coherence Tomography. Investig. Ophthalmol. Vis. Sci..

[24] Jung J.J., Chen C.Y., Mrejen S., Gallego-Pinazo R., Xu L., Marsiglia M., Boddu S., Freund K.B. (2014). The Incidence of Neovascular Subtypes in Newly Diagnosed Neovascular Age-Related Macular Degeneration. Am. J. Ophthalmol..

[25] Dansingani K.K., Tan A.C., Gilani F., Phasukkijwatana N., Novais E., Querques L., Waheed N.K., Duker J.S., Querques G., Yannuzzi L.A. (2016). Subretinal Hyperreflective Material Imaged with Optical Coherence Tomography Angiography. Am. J. Ophthalmol..

[26] Nassisi M., Lei J., Abdelfattah N.S., Karamat A., Balasubramanian S., Fan W., Uji A., Marion K.M., Baker K., Huang X. (2019). OCT Risk Factors for Development of Late Age-Related Macular Degeneration in the Fellow Eyes of Patients Enrolled in the HARBOR Study. Ophthalmology.

[27] Borrelli E., Battista M., Sacconi R., Gelormini F., Querques L., Grosso D., Vella G., Bandello F., Querques G. (2021). OCT Risk Factors for 3-Year Development of Macular Complications in Eyes With “Resolved” Chronic Central Serous Chorioretinopathy. Am. J. Ophthalmol..

[28] Guymer R.H., Markey C.M., McAllister I.L., Gillies M.C., Hunyor A.P., Arnold J.J., Chang A., Syed A., Broadhead G., Pham T. (2019). Tolerating Subretinal Fluid in Neovascular Age-Related Macular Degeneration Treated with Ranibizumab Using a Treat-and-Extend Regimen. Ophthalmology.

[29] Pokroy R., Mimouni M., Barayev E., Segev F., Geffen N., Nemet A.Y., Segal O. (2018). Prognostic value of subretinal hyperreflective material in neovascular age-related macular degeneration treated with bevacizumab. Retina.

[30] Sacconi R., Borrelli E., Vella G., Querques L., Prascina F., Zucchiatti I., Bandello F., Querques G. (2020). TriPla Regimen: A new treatment approach for patients with neovascular age-related macular degeneration in the COVID-19 “era”. Eur. J. Ophthalmol..

[31] Borrelli E., Grosso D., Vella G., Sacconi R., Battista M., Querques L., Zucchiatti I., Prascina F., Bandello F., Querques G. (2020). Short-term outcomes of patients with neovascular exudative AMD: The effect of COVID-19 pandemic. Graefes Arch. Clin. Exp. Ophthalmol..

[32] Holz F.G., Tadayoni R., Beatty S., Berger A., Cereda M.G., Cortez R., Hoyng C.B., Hykin P., Staurenghi G., Heldner S. (2015). Multi-country real-life experience of anti-vascular endothelial growth factor therapy for wet age-related macular degeneration. Br. J. Ophthalmol..

[33] Muether P.S., Hoerster R., Hermann M.M., Kirchhof B., Fauser S. (2012). Long-term effects of ranibizumab treatment delay in neovascular age-related macular degeneration. Graefes Arch. Clin. Exp. Ophthalmol..

[34] Rauch R., Weingessel B., Maca S.M., Vecsei-Marlovits P.V. (2012). Time to first treatment: The significance of early treatment of exudative age-related macular degeneration. Retina.

[35] Chew J.K., Broadhead G.K., Luo K., Hong T., Chang A.A., Zhu M. (2017). Bilateral Neovascular Age-Related Macular Degeneration: Comparisons between First and Second Eyes. Ophthalmology.

[36] Zarranz-Ventura J., Liew G., Johnston R.L., Xing W., Akerele T., McKibbin M., Downey L., Natha S., Chakravarthy U., Bailey C. (2014). The Neovascular Age-Related Macular Degeneration Database. Ophthalmology.

[37] Charafeddin W., Nittala M.G., Oregon A., Sadda S.R. (2015). Relationship Between Subretinal Hyperreflective Material Reflectivity and Volume in Patients With Neovascular Age-Related Macular Degeneration Following Anti-Vascular Endothelial Growth Factor Treatment. Ophthalmic Surg. Lasers Imaging Retin..

[38] Querques L., Parravano M., Borrelli E., Chiaravalloti A., Tedeschi M., Sacconi R., Zucchiatti I., Bandello F., Querques G. (2019). Anatomical and functional changes in neovascular AMD in remission: Comparison of fibrocellular and fibrovascular phenotypes. Br. J. Ophthalmol..

[39] Ores R., Puche N., Querques G., Blanco-Garavito R., Merle B.M., Coscas G., Oubraham H., Semoun O., Souied E.H. (2014). Gray Hyper-Reflective Subretinal Exudative Lesions in Exudative Age-Related Macular Degeneration. Am. J. Ophthalmol..

[40] Casalino G., Bandello F., Chakravarthy U. (2016). Changes in Neovascular Lesion Hyperreflectivity After Anti-VEGF Treatment in Age-Related Macular Degeneration: An Integrated Multimodal Imaging Analysis. Investig. Ophthalmol. Vis. Sci..

[41] Kumar J.B., Stinnett S., Han J.I.L., Jaffe G.J. (2020). Correlation of subretinal hyperreflective material morphology and visual acuity in neovascular age-related macular degeneration. Retina.

